# Evaluation of the Relationship between Presternal Fatty Tissue Thickness, Epicardial Fatty Tissue Volume, and Coronary Artery Disease

**DOI:** 10.2174/0115734056362293250712131404

**Published:** 2025-07-23

**Authors:** Turgut Tursem Tokmak

**Affiliations:** 1 Department of Radiology, Kayseri City Training and Research Hospital, Turkiye

**Keywords:** Epicardial adipose tissue, Sternum, Coronary artery disease, Heart, CT angiography, EFV

## Abstract

**Introduction:**

This cross-sectional study aimed to evaluate the relationship between presternal adipose tissue thickness and the pericardial adipose tissue volume in relation to coronary artery disease.

**Methods:**

A total of 108 patients who underwent coronary computed tomography angiography (CCTA) for suspected coronary disease between 2019 and 2022 were evaluated. Patients whose epicardial adipose tissue could not be optimally measured due to imaging artifacts, those with a pre-existing coronary artery anomaly or known heart disease, individuals under 18 years of age, and those who had undergone sternotomy and bypass surgery were excluded from the study. Accordingly, 95 patients (61 males and 34 females) who met the inclusion criteria and did not meet any of the exclusion criteria were included in the study. CCTA images were analyzed retrospectively. Pericardial adipose tissue volume was measured automatically using Syngo *Via* software. Presternal fat thickness (PFTT) was measured at the level of the pulmonary artery bifurcation, from the anterior to the posterior surface.

**Results:**

The study sample comprised 64.2% males and 35.7% females. The median thickness of the presternal fat tissue was found to be 11.5 mm, with a range of 3 to 44 mm. The median PFTT was measured at 9 mm (3−23 mm) in the male patient group, while in the female patient group, it was 20 mm (10−44 mm). The median epicardial fat volume (EFV) for the full sample was 83.1 ml (22.3−171 ml), measuring 81.1 ml (37−171 ml) and 79.5 ml (22.3−167 ml) in males and females, respectively. A significant correlation was observed between PFTT and EFV in the full sample (Rho = 0.236, p = 0.02), as well as among male patients (Rho = 0.409, p = 0.001), but not in the female patient group (Rho = 0.264, p = 0.131). In the male cohort, there was no significant difference between EFV and PFTT, and the presence of coronary plaque.

**Discussion:**

This study examines the relationship between presternal adipose tissue thickness (PFTT) and coronary artery disease (CAD), building on previous evidence that links epicardial adipose tissue (EAT) to cardiovascular risk. We found a significant correlation between PFTT and epicardial fat volume (EFV) in male patients, but not in females, which is likely due to hormonal influences and variability in breast tissue. Importantly, measurement of PFTT provides a practical, non-invasive method for assessing CAD risk in clinical settings. Although our small sample size limits the generalisability of our findings, these results suggest that PFTT may serve as an indirect marker of CAD risk and highlight the need for further research with larger cohorts to validate its clinical relevance. Incorporating PFTT assessment into routine practice may improve the early identification of high-risk patients and enhance strategies for preventing ischemic heart disease.

**Conclusion:**

The study reveals that increased presternal fat thickness correlates with elevated epicardial fat volume, indicating that presternal fat measurements could serve as a simple and effective tool for assessing the risk of coronary artery disease, particularly in male patients.

## 1 INTRODUCTION

Ischemic heart disease (IHD) is a significant global health concern, accounting for approximately 17 million deaths annually, making it the leading cause of mortality worldwide [1]. Early diagnosis and treatment of IHD are vital for preventing further complications and improving patient outcomes [2]. Identifying patients at low and intermediate risk for coronary artery disease (CAD) is essential for minimizing unnecessary invasive procedures and reducing healthcare costs.

Research has established a robust relationship between increased epicardial adipose tissue (EAT) and the development of coronary artery disease. EAT is known to trigger inflammation, elevate adiponectin secretion, and reduce thermogenic function, all of which contribute to the progression of atherosclerosis [3-5]. Recent advancements in cardiac computed tomography (CT) imaging have enabled precise measurement of EAT volume, highlighting its significant correlation with CAD [6-8]. Despite this knowledge, substantial research gaps persist, particularly regarding the relationship between presternal subcutaneous fat thickness (PFTT) and CAD outcomes.

Current literature predominantly emphasizes EAT while largely overlooking PFTT as a potentially important predictor of coronary artery disease [9, 10]. The correlation between PFTT and EAT volume has not been thoroughly investigated, and the implications of PFTT for the emergence of coronary artery lesions remain poorly understood. This gap represents a critical unknown in the assessment of CAD risk, highlighting the importance of understanding how these easily measurable parameters interact and contribute to cardiovascular health [11, 12].

This gap has motivated the present study, as part of which the relationship between presternal fat tissue thickness (PFTT) and epicardial fat volume (EFV) was investigated. Based on the obtained findings, we propose the adoption of PFTT as a practical, non-invasive tool for predicting CAD risk. By elucidating the correlation between PFTT and EFV, as well as the link between PFTT/EFV and the presence of coronary artery lesions, this research provides vital insights that contribute to enhanced risk assessment strategies, ultimately improving early intervention approaches in clinical practice. Such findings will fill a significant void in the existing literature, offering a novel perspective on the utility of presternal fat measurements in evaluating cardiovascular risk [13].

## 2 PATİENTS AND METHODS

After obtaining ethical approval from the Ethics Committee of Kayseri City Training and Research Hospital, a total of 108 patients who underwent coronary CT for suspected coronary disease between 2019 and 2022 were evaluated. Patients whose epicardial adipose tissue could not be optimally measured due to imaging artifacts, those with a pre-existing coronary artery anomaly or known heart disease, individuals under 18 years of age, and those who had undergone sternotomy and bypass surgery were excluded from the study. The inclusion criteria for this study consisted of patients aged 18 years and older who underwent coronary CT imaging for suspected coronary artery disease. Participants were required to have satisfactory epicardial adipose tissue measurements with minimal imaging artifacts. Additionally, individuals with no history of pre-existing coronary artery anomalies or known heart disease were included. A total of 13 patients were excluded from the study based on the exclusion criteria, while 95 patients were included in the study. Informed consent to participate in the study was obtained from all participants.

Sixty-one males and thirty-four females who met the inclusion criteria were enrolled in this study. The sample size was calculated based on an estimated effect size derived from previous studies, aiming for a statistical power of 80% to detect a significant correlation between presternal fat tissue thickness and epicardial fat volume. Based on a desired alpha level of 0.05, the calculated sample size was deemed sufficient to ensure robust findings. This approach helps mitigate the risk of Type I and Type II errors, thereby strengthening the validity of the results obtained in this study. The SAGER guidelines were followed to ensure equity in sex and gender throughout the research. Sex was determined based on patient self-report and confirmed *via* review of medical records. Gender was not specifically assessed in this study, and the terms 'sex' and 'gender' are used synonymously in this manuscript to refer to biological sex assigned at birth.

Sex was considered in the study design to ensure adequate representation of both males and females, reflecting the known differences in cardiovascular disease presentation and risk factors between sexes. While we did not implement specific sex- or gender-based interventions, we performed separate analyses for male and female cohorts to identify potential differences in the relationship between presternal fat thickness, epicardial fat volume, and coronary artery disease. No participants were excluded based on their sex/gender; the exclusion criteria were applied equally to both sexes.

**Fig. (1):**
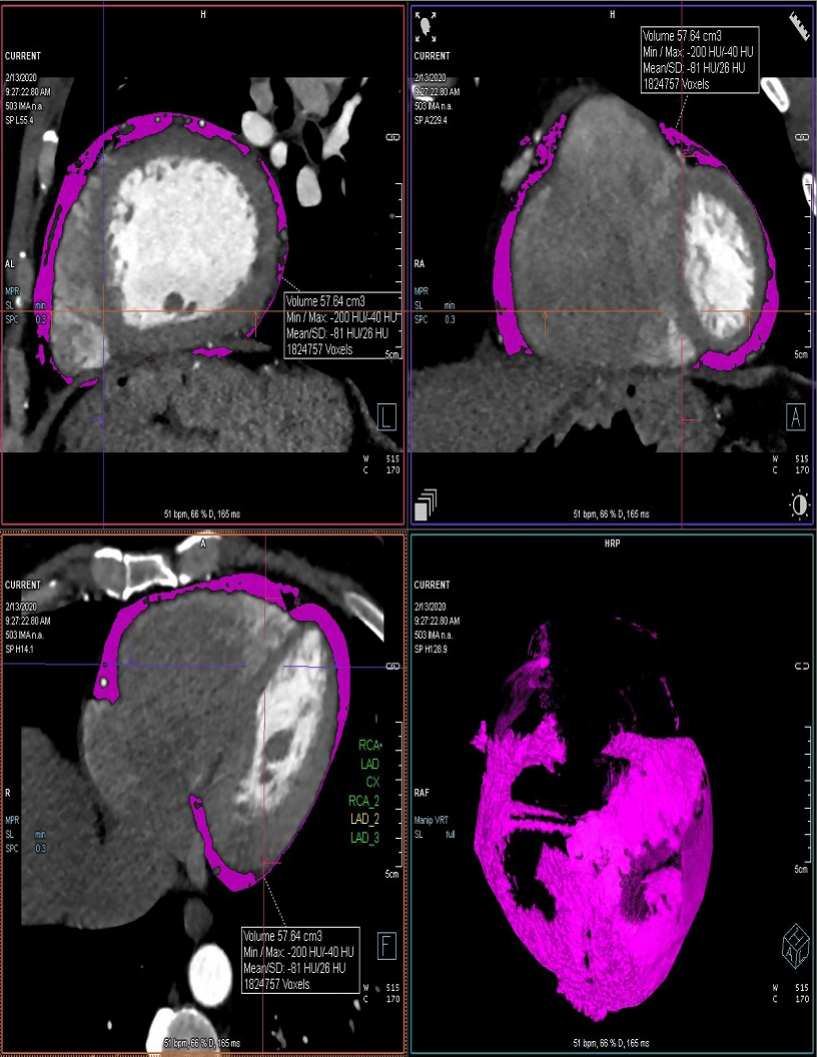
The volume of epicardial adipose tissue was measured automatically using density ranges between -30 HU and -220 HU.

**Fig. (2):**
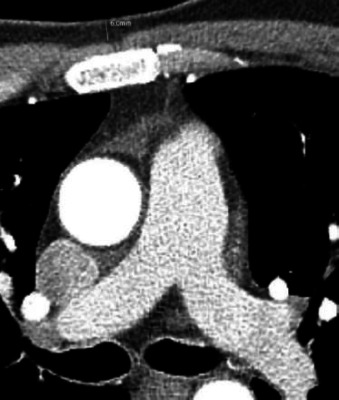
Presternal fat thickness was measured from anterior to posterior at the level of the pulmonary artery bifurcation on axial CT image.

Coronary CT images were analyzed retrospectively. The volume of epicardial adipose tissue was measured automatically using Syngo *Via* software (syngo.via, Siemens Healthcare Sector, Forchheim, Germany) with density ranges between -30 HU and -220 HU (Fig. **1**). Presternal fat thickness was measured from anterior to posterior at the level of the pulmonary artery bifurcation (Fig. **2**). Coronary artery lesions were evaluated by a radiologist with 10 years of experience and classified as normal coronary arteries, plaques without significant stenosis (<50%), and plaques with significant stenosis (>50%). The coronary artery calcium score was determined automatically using Syngo *Via* software (syngo.via, Siemens Healthcare Sector, Forchheim, Germany).

Prior to the CT procedure, all patients received 100 mg of a beta-blocker to maintain an optimal heart rate (60-70 beats per minute) on the day before and the day of the procedure. All patients underwent CTA using a 128-slice multidetector CT (SOMATOM Definition Edge, Siemens Medical Solutions, Erlangen, Germany). The CT scan was acquired with the following settings: collimation of 0.6 mm, slice acquisition of 2 x 128 x 0.6 mm using an x-flying focal spot, pitch of 0.2, tube voltage of 100-120 kV, and gantry rotation time of 330 ms. Patients were administered a bolus of 80 mL of contrast medium (Iohexol 350 mosm). All coronary CTA acquisitions were obtained using a retrospective electrocardiographic gating method. The CTA data were transferred to a post-processing workstation (Syngo via, Siemens Healthcare Sector, Forchheim, Germany). The threshold values of the automatic software package were adjusted between -30 HU and -220 HU and remained constant for all patients. Data were analyzed using SPSS for Windows (version 23). Non-parametric tests (Spearman’s rho, etc.) were used for the statistical analysis.

## 3 RESULTS

A total of 95 patients were included in the study, comprising 61 males (64.2%) and 34 females (35.8%). The age range of the male patients was between 27 and 72 years, while that of the female patients was between 31 and 79 years. The mean age of the male patient cohort was 53.3 ± 10.7 years, whereas the female patient group had a mean age of 56.4 ± 11.1 years (Table **1**).

The median thickness of the presternal fat tissue was found to be 11.5 mm, with a range of 3 to 44 mm. In the male patient group, the median PFTT was measured at 9 mm, with a range of 3 to 23 mm. In contrast, the median PFTT for the female patient group was 20 mm, with a range of 10 to 44 mm. The median epicardial fat volume (EFV) was determined to be 83.1 mL, with a range of 22.3 mL to 171 mL. Specifically, the median EFV for the male patient group was 81.1 ml, ranging from 37 ml to 171 ml, while in the female group, the median EFV was 79.5 ml, with a range of 22.3 ml to 167 ml (Table **1**).

We present comprehensive baseline characteristics of our patient population, separated by gender. In the male participants (n = 61), the mean body mass index (BMI) was 29.7 kg/m^2^, with a range of 28 to 33 kg/m^2^, indicating a prevalence of overweight and mildly obese individuals. The average low-density lipoprotein (LDL) cholesterol level in males was 137.3 mg/dL (range, 120-160 mg/dL). In comparison, the average high-density lipoprotein (HDL) cholesterol level was 43.1 mg/dL (range, 36-50 mg/dL). Additionally, triglyceride levels averaged 157.7 mg/dL, with a range of 134 to 190 mg/dL (Table **2**).

In contrast, the female participants (n = 34) had a mean BMI of 27.9 kg/m^2^, indicating a similar demographic of overweight individuals but with lower mean values compared to males. The average LDL cholesterol level among females was 128.9 mg/dL (range: 115 to 140 mg/dL), whereas the average HDL cholesterol was slightly higher at 48.3 mg/dL (range: 44 to 55 mg/dL). Triglyceride levels for females averaged 140.7 mg/dL, with a range of 125 to 155 mg/dL (Table **3**).

**Fig. (3):**
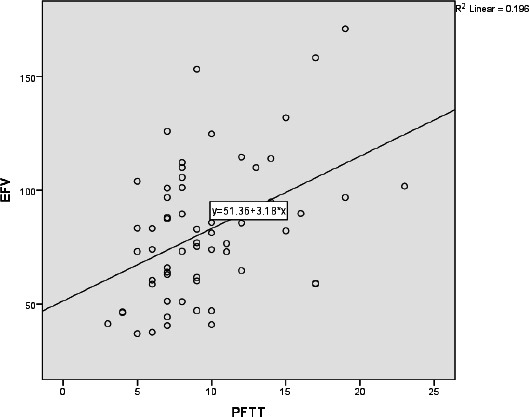
Scatter plot illustrating the relationship between PFTT and EFV in male participants.

**Fig. (4):**
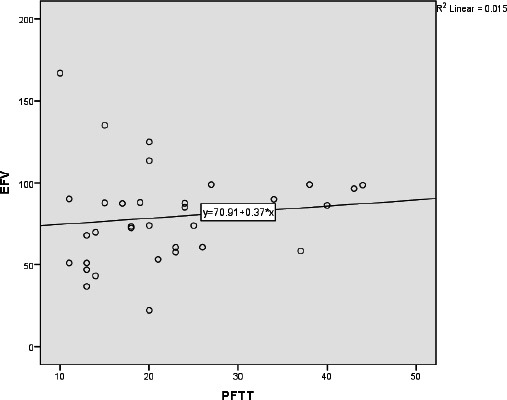
Scatter plot illustrating the relationship between PFTT and EFV in female participants.

A significant correlation was found between PFTT and EFV (Rho = 0.236, p = 0.02) in all patients. When the male and female patient groups were evaluated separately, a statistically significant positive correlation was found between PFTT and EFV in the male patient group (Rho = 0.409, p = 0.001) (Fig. **3**). However, no significant correlation was observed in the female patient group (Rho = 0.264, p = 0.131) (Fig. **4**).

The coronary artery calcium score ranged from 0 to 537, with a median score of 2.5. In the male patient group, the calcium score ranged from 0 to 537, while in the female patient group, it ranged from 0 to 165, with median calcium scores of 6.5 and 0, respectively. No significant correlation was found between epicardial fat volume, presternal fat thickness, and coronary artery calcium score in both patient groups (Rho = 0.264, p = 0.131).

In total, 39 patients (22 males and 17 females) had no plaque or stenosis in the coronary arteries. Forty-one patients (28 males and 13 females) exhibited plaque formation without significant stenosis, and 15 patients (10 males and 5 females) had significant stenosis. No significant correlation was observed between epicardial fat volume, presternal fat thickness, and the presence of coronary artery stenosis in the male group. However, a significant correlation was detected between epicardial fat volume and coronary artery stenosis in the female group (Rho = 0.465, p = 0.006).

**Fig. (5):**
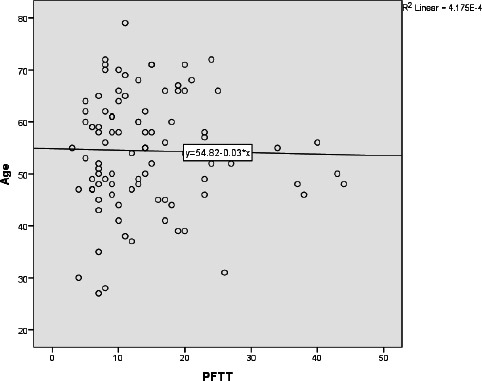
Scatter plot illustrating the relationship between Presternal Fat Tissue Thickness (PFTT) and the ages of the participants.

**Fig. (6):**
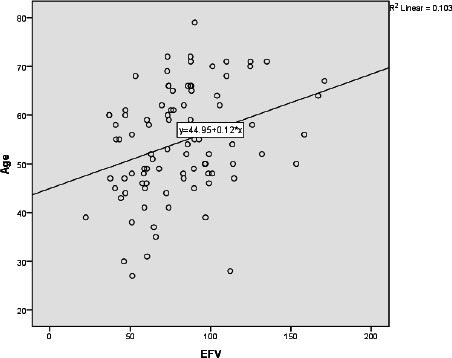
Scatter plot illustrating the relationship between Epicardial Fat tissue Volume (EFV) and the ages of the participants.

No significant correlation was found between presternal fat tissue thickness (PFTT) and age (Fig. **5**). When comparing the age of patients with the epicardial fat volume (EFV), a statistically significant increase in adipose tissue volume was observed with increasing age (Rho = 0.345, p = 0.001) (Fig. **6**). Additionally, a statistically significant correlation was observed between patient age and the coronary artery calcium score, as well as the presence of calcified plaque (Rho = 0.486, p = 0.001).

No statistically significant correlation was found between the coronary artery calcium score and EFV in either group. Furthermore, there was no significant difference between EFV and the presence of coronary plaque in the male patient group. The PFTT also showed no significant difference when compared with the presence of coronary artery plaque in the male patient group.

## 4 DISCUSSION

Given the prevalence of IHD, its early diagnosis and treatment are crucial to prevent further deterioration in patients’ health. Increased epicardial adipose tissue has been shown to trigger coronary heart disease by enhancing the release of free fatty acids and cytokines [3-5]. Numerous studies have demonstrated that increases in epicardial adipose tissue and visceral abdominal adipose tissue correlate with an elevated CAD risk [6-8].

Despite the established link between epicardial adipose tissue and CAD, its relationship with presternal adipose tissue has not been directly investigated. However, as a part of their recent study, Topcu and colleagues found a significant association between increased thickness of presternal adipose tissue and epicardial adipose tissue with the severity of COVID-19 [10]. In our study, we aimed to investigate whether an increase in presternal fat could also serve as an indicator for the risk of coronary heart disease.

A strong association has been consistently observed between coronary heart disease and both epicardial fat thickness and volume [11-14]. Measurements of epicardial adipose tissue thickness can be obtained *via* echocardiography, whereas volume can be assessed using cardiac CT [15, 16]. However, both methods necessitate specialized equipment and expertise. In contrast, as presternal fat is located just above the breastbone and is situated subcutaneously, it can be measured manually during clinical examinations with skinfold calipers, representing a practical and straightforward assessment method [17].

In our study, the pulmonary artery bifurcation was chosen as the anatomical landmark for measuring PFTT because it provides a consistent and reproducible reference point in the thoracic cavity, minimizing variability resulting from individual anatomical differences while ensuring a representative assessment of subcutaneous fat located just above the breastbone. Alternative anatomical landmarks were considered for measuring PFTT, such as the xiphoid process and the aortic arch; however, these were found to either introduce greater variability in measurements or be less consistently visible across different patient anatomies in our imaging protocols [18]. The pulmonary artery bifurcation provides a mid-sternal reference that strikes a balance between accessibility and reproducibility, making it an optimal choice for our study.

We found no statistically significant relationship between PFTT and EFV in the female patient group, which may be attributed to the fatty tissue variability in female patients’ breasts and possibly to female-specific obesity patterns. Age and estrogen deficiency significantly influence the observed outcomes. As women age, particularly post-menopause, the decline in estrogen levels is associated with increased visceral fat accumulation and changes in fat distribution [12, 19, 20]. This hormonal change can adversely affect lipid metabolism and vascular function, potentially contributing to increased cardiovascular risk [14, 21]. Conversely, there was a significant association between EFV and the presence of coronary lesions in the female cohort.

In the male patient group, a statistically significant correlation was found between PFTT and EFV; however, no significant association emerged between EFV and the presence of coronary lesions. We posit that this outcome may be due to the limited number of patients with significant coronary lesions, as well as the fact that these patients were primarily categorized within low to intermediate risk groups. Given the findings published by Si *et al*. indicating that the increase in presternal adiposity among men correlates with increased epicardial adiposity, presternal adiposity may be an indirect marker of coronary heart disease risk [22].

Importantly, we need to address the limitations of our study, particularly the small sample size and the limited number of female patients, which preclude the generalizability of our findings beyond the studied cohort. This limitation underscores the need for future studies using larger and more diverse samples, thereby enabling researchers to validate these findings. Increasing the sample size will enhance our understanding of the relationships among presternal fat thickness, epicardial fat volume, and coronary artery disease, as well as their effects in different demographic groups, thereby improving the accuracy of risk assessment in clinical practice.

Extant studies have also linked higher body fat percentages to various cardiometabolic diseases, including hypertension, type 2 diabetes, and dyslipidemia, suggesting that excess body fat can exert detrimental effects on cardiovascular health [23-25]. For instance, elevated body fat percentage is associated with an increased risk of developing metabolic syndrome, which is a significant predictor of cardiovascular events [11]. Moreover, older age is a well-recognized risk factor for CAD due to cumulative risk exposures over time, including hypertension, diabetes, and lifestyle factors [26]. Body fat distribution is important even in individuals of normal weight, as it can increase the risk of cardiovascular disease, regardless of body weight [27]. These demographic factors can further influence the relationship between fat distributions (such as presternal and epicardial adipose tissues) and coronary disease risk, underscoring the need to consider these variables in future research [12].

Given these findings, measuring presternal adipose tissue thickness serves as a practical and straightforward approach that can be incorporated into routine clinical examinations. Male patients with measurements above specific threshold values should be referred for further evaluation, as early detection of high-risk patients can significantly mitigate potential cardiac damage stemming from ischemic heart disease [28]. Ultimately, expanding our understanding of the relationship between presternal fat measurements and cardiovascular health can lead to more effective prevention strategies and intervention methods for coronary artery disease.

## 5 STUDY LIMITATIONS

This study has several limitations that should be acknowledged. First, its retrospective design may introduce biases in selection and data collection, impacting the reliability of the obtained findings. The relatively small sample size, particularly a small number of female patients, limits the generalizability of our results and may have contributed to the lack of significant associations observed in this group.

Second, the use of cardiac CT involves radiation exposure, which raises safety concerns and requires specialized equipment and expertise, potentially restricting its routine clinical application. Third, our study does not account for temporal changes in body composition resulting from age and hormonal fluctuations, suggesting that longitudinal studies are necessary for a more comprehensive understanding.

Finally, although we identified correlations between presternal fat thickness and epicardial fat volume, causality cannot be established from our findings. Therefore, further investigations are necessary to validate these results in larger and more diverse populations.

## CONCLUSION

This study’s findings suggest that increased presternal fat thickness could serve as a practical, non-invasive marker for assessing coronary heart disease risk in male patients. Recognizing higher levels of presternal fat may prompt closer monitoring and the implementation of preventive strategies in clinical settings.

## Figures and Tables

**Table 1:** Clinical characteristics of patients, evaluation, and measurements.

**Clinical Characteristic of Patients**	**Male Group**	**Female Group**	**All Patients**
Number	61 (64.2%)	34 (35.7%)	95 (100%)
Average age	53.3±10.7	56.4 ±11.1	54.7±11.1
Median presternal fat tissue thickness (PFTT)	9 mm (3-23)	20mm (10-44)	11.5mm (3-44)
Epicardial fatty tissue volume	81.1 ml (37-171)	79.5 ml (22.3-167)	81.3 ml (22.3-171)
Coronary artery calcium score	6.5 (0-537)	0 (0-165)	2.5 (0-537)
Without coronary plaque	22	17	39 (41%)
Coronary plaque with no significant stenosis	28	13	41 (43%)
**Coronary plaque with significant stenosis**	**10**	**5**	**15 (16%)**

**Table 2:** This table summarizes the key metrics for the participants, including Body Mass Index (BMI), Low-Density Lipoprotein (LDL) cholesterol levels, High-Density Lipoprotein (HDL) cholesterol levels, and triglyceride levels. The table presents the sample size (N), minimum and maximum values, mean values, and standard deviations for each characteristic, providing insights into the health profiles of the male participants in this study.

**Descriptive Statistics of Baseline Characteristics and Lipid Profile in Male Participants**					
	**N**	**Minimum**	**Maximum**	**Mean**	**Std. Deviation**
**BMI (kg/m^2^)**	61	28	33	29.71	1.127
**LDL (mg/dL)**	61	120	160	137.38	10.325
**HDL (mg/dL)**	61	36	50	43.10	3.400
**Triglycerides (mg/dL)**	61	134	190	157.77	12.039
**Valid N (listwise)**	61				

**Table 3:** This table summarizes the key metrics for the participants, including Body Mass Index (BMI), Low-Density Lipoprotein (LDL) cholesterol levels, High-Density Lipoprotein (HDL) cholesterol levels, and triglyceride levels. The table presents the sample size (N), minimum and maximum values, mean values, and standard deviations for each characteristic, providing insights into the health profiles of the female participants in this study.

**Descriptive Statistics of Baseline Characteristics and Lipid Profile in Female Participants**					
	**N**	**Minimum**	**Maximum**	**Mean**	**Std. Deviation**
**BMI**	34	26	30	27.90	1.075
**LDL (mg/dL)**	34	115	140	128.91	7.038
**HDL (mg/dL)**	34	44	55	48.38	2.934
**Triglycerides (mg/dL)**	34	125	155	140.76	8.777
**Valid N (listwise)**	34				

## Data Availability

All data generated or analyzed during this study are included in this published article.

## LIST OF ABBREVIATIONS

IHD: Ischemic Heart Disease
**CT**: Computerized Tomography
CCTA: Coronary Computed Tomography Angiography
CAD: Coronary Artery Disease
EFV: Epicardial Fat Volume
PFTT: Presternal Fat Tissue Thickness
EAT: Epicardial adipose tissue
BMI: Body Mass Index
LDL: Low-Density Lipoprotein
HDL: High-Density Lipoprotein

## STANDARDS OF REPORTING

STROBE guidelines were followed.
